# Temperature and Precipitation Jointly Shape the Plant Microbiome by Regulating the Start of the Growing Season

**DOI:** 10.1111/gcb.70431

**Published:** 2025-08-14

**Authors:** Dina in ’t Zandt, Anna Florianová, Mária Šurinová, Michiel H. in ’t Zandt, Kari Klanderud, Vigdis Vandvik, Zuzana Münzbergová

**Affiliations:** ^1^ Institute of Botany Czech Academy of Sciences Průhonice Czech Republic; ^2^ Department of Botany, Faculty of Science Charles University Prague Czech Republic; ^3^ Soil Biology Group Wageningen University and Research Wageningen the Netherlands; ^4^ Faculty of Environmental Sciences and Natural Resource Management Norwegian University of Life Sciences Ås Norway; ^5^ Department of Biological Sciences and Bjerknes Centre for Climate Research University of Bergen Bergen Norway

**Keywords:** microbial co‐occurrence networks, precipitation, rhizosphere microbiome, root microbiome, seasonality, snow cover, temperature

## Abstract

Climate change is altering associations between plants and soil microbiota, threatening ecosystem functioning and stability. Predicting these effects requires understanding how concomitant changes in temperature and precipitation influence plant–soil microbiota associations. We identify the pathways via which temperature and precipitation shape prokaryote and fungal rhizosphere and root‐associated networks of the perennial grass *Festuca rubra* in cold‐climate ecosystems. We found that joint effects of temperature and precipitation are key in shaping plant–soil microbiota associations, with the start of the growing season as a critical mediating factor. Specifically, the start of the growing season is advanced by increasing temperature but delayed by increasing precipitation. This joint pathway particularly shaped rhizosphere organic matter degrading microbiota and root‐associated putative plant pathotroph‐saprotrophs and beneficial microbiota. We conclude that understanding local temperature, precipitation, and seasonal changes is crucial to accurately predict how the unique plant‐microbiota interactions shaping cold‐climate ecosystems are evolving with the ongoing change in climate.

## Graphical abstract

We studied how temperature and precipitation affect the soil and root‐associated microbes of a common grass species in cold climates. Our results show that these climate factors jointly shape the microbial communities plants interact with. These effects largely stem from shifts in the start of the growing season, with warmer temperatures advancing and higher precipitation delaying its onset.

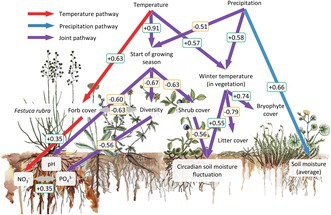

## 1 Introduction

The ongoing changes in climate are altering interactions between plants and soil microbiota. Established interactions are being disrupted, while novel interactions emerge (Bardgett and Caruso 2020). These shifts in plant–soil microbiota interactions pose a threat to the functioning and stability of ecosystems, as these interactions are a critical driver of numerous plant community processes, including succession, coexistence, invasion, range expansion, diversity, and stability (Aldorfová et al. 2020; Bever et al. 2012; Engelkes et al. 2008; in 't Zandt et al. 2021, 2022; Kardol et al. 2006; Semchenko et al. 2019; van der Putten et al. 2016). Climate change impacts are particularly concerning in cold‐climate ecosystems, where warming is occurring faster than the global average, and biological processes are often limited by low temperatures (IPCC 2021). As these ecosystems warm, the conditions that plant–soil microbiota interactions are adapted to are lifted, likely resulting in profound consequences for overall ecosystem functioning (Florianová and Münzbergová 2025).

At the same time, precipitation patterns in cold‐climate regions are changing. These concurrent shifts in precipitation and temperature are likely to have complex effects on plant–soil‐microbiota interactions (IPCC 2021). However, studies often focus on a single climate factor at a time, leaving a significant gap in our understanding of how simultaneous changes in temperature and precipitation impact these interactions. This knowledge gap limits our ability to predict the broader consequences of climate change on cold‐climate ecosystem functioning and stability. In this study, we fill this knowledge gap by identifying how concomitant changes in temperature and precipitation shape plant–soil microbiota interactions in cold regions.

Interactions between plants and soil microbiota occur in the rhizosphere, the thin layer of soil surrounding plant roots, as well as on the root surface (rhizoplane) and inside the root (endosphere) (Dini‐Andreote 2020; Philippot et al. 2013). These interactions are sensitive to climate change, as even minor climatic changes trigger rapid physiological responses in plants and strong alterations in soil microbial community composition (Bardgett and Caruso 2020; Trivedi et al. 2022). While the effects of warming on plant–soil microbiota interactions in cold regions are relatively well understood, the role of precipitation and its combined effect with warming has received considerably less attention. This is largely because studies typically focus on the presumed limiting factor of a system, such as temperature in cold climates and precipitation in dry regions. Additionally, precipitation is inherently more complex than temperature because it can fall as either rain or snow. This means that the consequences of changing precipitation patterns depend on when in the season precipitation changes, and thus whether snowfall or rainfall is affected, but also whether temperature changes concomitantly, potentially altering the form of precipitation. Not only do temperature and precipitation jointly determine soil moisture, their joint effects also determine snow depth, the timing of snowmelt, and thus the onset of the growing season in cold‐climate ecosystems. Given this complexity, understanding how simultaneous changes in both temperature and precipitation affect plant–soil microbiota interactions is essential for predicting impacts of climate change on cold‐climate ecosystems.

In cold‐climate ecosystems, warming raises the metabolic activity of both plants and soil microbiota. In plants, warming increases photosynthetic activity, leading to greater carbon (C) allocation belowground through increased root growth and root exudation (Bengtson et al. 2012; Ferrari et al. 2018; Medlyn et al. 2002; Wang et al. 2019; Zhang et al. 2020). This increase in belowground C allocation affects plant interactions with soil microbiota by, for example, enhancing rhizosphere priming: the promotion of rhizosphere microbiota performing mineralisation to increase nitrogen (N) availability (Bengtson et al. 2012; Keuper et al. 2020; Zhu and Cheng 2011). In soil microbial communities, a rise in nutrient availability favours fast‐growing microbiota over slow‐growing microbiota (Leff et al. 2015). This shift typically results in microbial communities becoming increasingly dominated by faster‐growing prokaryotes compared to the generally slower‐growing fungi (Wang and Kuzyakov 2024a). A dominance of fast‐growing microbiota is associated with accelerated soil C and N cycling, resulting from increased soil organic matter turnover with warming (Crowther et al. 2016; Ferrari et al. 2018; Kirschbaum 2000). Consequently, warming of cold‐climate ecosystems impacts both plant and microbial performance individually.

However, with changing precipitation, warming effects on plant–soil microbiota interactions are also likely to change (Florianová and Münzbergová 2025). With the overall low metabolic activity and low evapotranspiration in cold conditions, precipitation increases soil moisture more significantly in cold than in warm conditions (Kirschbaum 2000). Consequently, in warm conditions, an increase in precipitation may fuel the higher metabolic rates of both plant and microbial communities, amplifying the effects of warming on plant, soil, and microbial processes. In contrast, in cold conditions, increased precipitation may lead to more frequent anoxic soil conditions, which severely impacts both plant and microbial performance. For example, soil anoxia reduces soil microbial respiration and decomposition rates and favors soil denitrification over nitrification pathways (Lin et al. 2021; Tiedje et al. 1984). In plants, soil anoxia reduces CO_2_ assimilation and nutrient uptake while also triggering anaerobic root metabolism and changes in root traits, such as root tissue density (Oram et al. 2020, 2021; Parent et al. 2008).

On a seasonal scale, changes in precipitation—specifically snowfall—are likely to affect how warming shapes plant–soil microbiota associations by influencing the start of the growing season. While an increase in temperature accelerates snowmelt and advances the start of the growing season, an increase in precipitation increases snow depth and delays the start of the growing season (Rixen et al. 2022). Snow removal and snow addition experiments demonstrate the key role that advances and delays in the start of the growing season play in shaping soil biogeochemical and soil microbial communities (Broadbent et al. 2021, 2024; Gavazov et al. 2017). In cold‐climate ecosystems, temperature and precipitation changes are thus likely to jointly reshape plant–soil microbiota associations. However, we currently lack a clear understanding of the mechanisms by which these factors jointly influence these associations.

Beyond direct effects of warming, precipitation, and their joint effect on plant–soil microbial associations, climate change in cold‐climate ecosystems is likely to extensively alter plant–soil microbiota associations through indirect pathways. These indirect pathways may include shifts in plant community composition, soil biogeochemical processes, and changes in the neighboring plant species with which host plants interact. For instance, warming of cold ecosystems increases plant community productivity, favoring fast‐growing plant species and leading to the exclusion of slow‐growing species, thereby decreasing plant community diversity (Klanderud et al. 2015; Vandvik et al. 2020). Such changes in plant community composition and diversity may, in turn, influence plant–soil microbiota associations by altering soil biogeochemical processes and modifying the neighboring species with which a host plant interacts (in 't Zandt et al. 2023). We currently lack insight into the extent to which direct versus indirect climate‐driven pathways mediate the effects of simultaneous changes in temperature and precipitation on plant–soil microbiota associations.

In this study, we test how prokaryote and fungal rhizosphere and root‐associated (rhizoplane and endosphere) communities of *Festuca rubra* in cold‐climate ecosystems are affected by temperature, precipitation, and their joint effect. *F. rubra* is a widely distributed perennial grass species growing in multiple climatic zones. This species therefore provides a unique opportunity of testing shifts in rhizosphere and root‐associated microbiomes along large climatic gradients. We sampled *F. rubra* in the Vestland Climate Grid located in the fjords of southern Norway (Klanderud et al. 2015, 2017). The grid comprises 12 natural grassland locations differing in average summer temperatures (6.5°C, 8.5°C and 10.5°C) and average annual precipitation (600, 1200, 2000 and 2700 mm) in a factorial design (Klanderud et al. 2015, 2017). We determined rhizosphere and root‐associated prokaryote and fungal communities of eight *F. rubra* individuals from each location using 16S and ITS amplicon sequencing. Additionally, we measured soil abiotic properties and determined aboveground plant community composition. We ask (i) what is the relative importance of temperature, precipitation, and their joint effects in shaping rhizosphere and root‐associated prokaryote and fungal community composition, (ii) via which pathways these effects occur: directly as a response to changes in climate and seasonality, or indirectly, mediated by changes in plant community composition and/or soil abiotic properties, and (iii) which soil microbial groups are reshaped by these climate‐driven pathways. Based on our findings, we discuss the effects of the ongoing concomitant changes in temperature and precipitation on plant–soil microbiota associations in cold‐climate ecosystems, and the implications these shifts are likely having on ecosystem functioning and stability.

## 2 Methods

### 2.1 Experimental Design

The sampling took place in the Vestland Climate Grid (Klanderud et al. 2015, 2017). The grid comprises 12 locations in the fjords of southern Norway with factorial combinations of three temperature levels (mean growing season temperatures of 6.5°C, 8.5°C and 10.5°C) and four precipitation levels (annual precipitation of 600, 1200, 2000 and 2700 mm) (Figure S1). These initial temperature and precipitation levels were calculated based on interpolation of meteorological data from 1961 to 1990 (Klanderud et al. 2015, 2017). Since 2009, each location in the grid has air and soil temperature sensors and soil moisture sensors installed, providing detailed climate information. All 12 locations are natural grassland ecosystems on a calcareous bedrock. The perennial, clonal grass *Festuca rubra* is the only plant species consistently growing at each location and hence was selected as the focal plant species in this study. In the same climate grid, *F. rubra* was found to show both genetic differentiation and plastic responses in its growth traits in relation to temperature, precipitation, and their interaction (Kosová et al. 2022; Münzbergová et al. 2017). These responses on the plant species level provide a strong basis to suggest consistent changes in the interactions of *F. rubra* with prokaryote and fungal communities within the climate grid as well.

### 2.2 Sampling

In July 2020, rhizosphere soil and roots of eight individuals of *F. rubra* at each location were collected. The eight individuals per location were selected from a line transect of individuals growing at least 1 m away from each other to avoid sampling of the same *F. rubra* clone. Rhizosphere soil and roots were sampled by taking a soil core of 5 cm in diameter and 5 cm depth at the spot of the *F. rubra* individual. In the field, the plant root system was carefully separated from the bulk soil, and only roots attached to the *F. rubra* shoot were collected. Rhizosphere soil was carefully separated from plant roots by brushing the thin layer of soil off the roots and stored at 4°C. Roots were washed in milliQ water to remove any remaining attached soil particles and loosely attached microbiota. Roots were lightly dried using a paper towel and stored on silica gel. Bulk soil for soil chemical analysis was collected from the soil core of each *F. rubra* individual and stored at 4°C. In the laboratory, both rhizosphere soil and root tissue were frozen at −80°C until further analyses.

### 2.3 Rhizosphere and Root Microbiome Amplicon Sequencing

Silicagel dried rhizosphere samples (250 mg each, in duplicates for each sample) and roots (20 mg each, in duplicates for each sample) were homogenized and lysed in PowerBead Tubes (Qiagen, Hilden, Germany) on a Vortex adapter. DNA was extracted using the DNeasy PowerSoil Kit (Qiagen, Germany) according to the manufacturer's instructions. The fungal internal transcribed spacer of the rRNA (ITS2) was amplified using primers gITS7ngs (Ihrmark et al. 2012) and ITS4 (White et al. 1990). The bacterial 16S rRNA gene (V4 region) was amplified from the same DNA extracts using primers 515f and 806r (Caporaso et al. 2011).

PCR amplification was designed in two subsequent reactions: the first PCR (PCR1) was performed with non‐barcoded primers, and the second (PCR2) with non‐barcoded primers tagged with sample‐specific barcodes. The product of the first PCR was used as a DNA template for the second PCR reaction. DNA amplification of fungal sequences was performed in 15 μL (PCR1) and 30 μL (PCR2) reactions as follows: PCR1: 1× PCR Blue Buffer wo MgCl_2_ (Top‐Bio, Vestec, Czech Republic), 2 mM MgCl_2_, 10 μg BSA, 0.2 mM each dNTP, 0.4 μM of each primer, 0.35 U *Taq* DNA Polymerase (Top‐Bio, Vestec, Czech Republic) and 20 ng of DNA dissolved in deionized water. PCR2: 1× PCR Blue, 2 mM MgCl_2_, 20 μg BSA, 0.2 mM each dNTP, 0.2 μM of each primer, 0.7 U *Taq* DNA Polymerase, and 2 μL of PCR1 product. The following thermocycler conditions were used: an initial denaturation step at 94°C for 5 min, followed by 35 cycles of denaturation (94°C for 30s), annealing (45°C for 30s for rhizosphere samples and 50°C for 30s for root samples) extension (72°C for 45 s), and the final extension at (72°C for 20 min). DNA amplification of bacterial sequences was performed in 15 μL (PCR1) and 30 μL (PCR2) reactions as follows: PCR1: 1× PCR Buffer, 2 mM MgCl_2_, 10 μg BSA, 0.2 mM each dNTP, 0.2 μM of each non‐barcoded primer, 0.35 U *Taq* DNA Polymerase, and 10 ng of DNA dissolved in deionized water; PCR2: 1× PCR Buffer, 2 mM MgCl_2_, 20 μg BSA, 0.2 mM each dNTP, 0.2 μM of each primer, 0.7 U *Taq* DNA Polymerase, and 1 μL of the PCR1 product.

Each DNA sample was extracted in technical duplicates. PCR1 was performed without technical multiplication, and PCR2 was performed in technical duplicates to capture maximum diversity from each sample. The pooled tetraplicates were purified by using the QIAquick PCR Purification Kit (Qiagen, Hilden, Germany) according to the manufacturer's protocol. DNA quantification was assessed by the Qubit 2.0 Fluorometer (Thermo Fisher Scientific). The purified amplicons were pooled in equimolar ratios. Two rhizosphere and two root negative PCR controls (ddH_2_O instead of a template) were included in the described workflow. The final amplicon library was sequenced on an Illumina MiSeq instrument (2 × 250 bp) (SEQme, Dobříš, Czech Republic).

### 2.4 16S and ITS Bioinformatics

In total, we sequenced 96 rhizosphere and 96 root samples with two negative controls each. Raw reads were processed using the pipeline SEED2 ver. 2.1.1b (Větrovský et al. 2018) in which reads were demultiplexed (no mismatch allowed in the tag sequences). Low‐quality sequences with a mean quality score < 30 were discarded, including reads with non‐matching tags. Primers and barcode sequences were cut from the reads.

All following bioinformatics on demultiplexed raw FASTQ files were analyzed using the DADA2 pipeline for prokaryote 16S and fungal ITS sequences in R version 4.1.2 (Callahan et al. 2016; R Core Team 2021). Both 16S and ITS sequences were quality trimmed and filtered according to the standard DADA2 protocols for 2 × 250 bp reads. Increasing the maxEE quality filter did not improve the quality filtering of ITS sequences (Rolling et al. 2022). After filtering and trimming, sequences were inferred based on a parametric error model, after which chimeric sequences and ASVs occurring in the blank samples were removed as these were likely contaminants. Taxonomic information was obtained using the SILVA (v138) and UNITE (v8.2) databases for 16S and ITS, respectively (Nilsson et al. 2019; Quast et al. 2013). Non‐prokaryote and non‐fungal hits were removed as well as mitochondrial and chloroplast hits. The procedure resulted in 35,067 unique 16S ASVs and 8881 unique ITS ASVs. These were represented by 4,098,759 and 4,611,230 total reads for the 16S and ITS amplicons, respectively. ASVs with in total < 100 reads were removed as well as one sample with in total < 1000 reads, and an outlier likely originating from a plant that was not *F. rubra* .

### 2.5 Microbial Community Calculations

Read counts were normalized using centered log ratio (clr) transformation and visualized using PCA with the phyloseq package (McMurdie and Holmes 2013). Significant separation along gradients of temperature, precipitation, and their interaction (continuous variables) was tested with PERMANOVA using *adonis* of the vegan package (Oksanen et al. 2018). PERMANOVA tests were performed on sample centroids per location to take the nested design of eight samples per location into account. To visualize these effects on the first two axes of the ordination, a passive overlay of significant climate predictors (temperature, precipitation and/or their interaction as continuous variables) was created using *envfit* of the vegan package, again using centroids per location to take the nested design into account (Oksanen et al. 2018). Shannon diversity was calculated based on multiple rarefaction (1000 iterations) using the metagMisc package (Mikryukov 2021).

For each ASV, we calculated a specialisation index (SI) for the rhizosphere and root associated compartments each following Chen et al. (2021). SI of the *i*th ASV was calculated as the coefficient of variation minus a correction for under‐sampling of rare ASVs:

$${\mathrm{SI}}_{i}=\left(\frac{\sigma_{i}}{\mu_{i}}\right)-\sqrt{\frac{K}{N_{i}}}$$

with $\sigma_{i}$ being the standard deviation of the reads of the *i*th ASV across all samples, $\mu_{i}$ being the mean of the reads of the *i*th ASV across all samples, K the number of habitat classes (12 locations) and N the total number of reads of the *i*th ASV across all samples. Calculations were based on rarefied read abundances, which was performed to the smallest sample sizes: 3766 reads for 16S and 1828 reads for ITS. The read numbers resulted from consistently lower sequencing depth in root samples compared to rhizosphere samples, rather than from a few outlier samples. Average SI of each sample was calculated as the community weighted mean of the SI of all ASVs present in the sample.

### 2.6 Microbial Co‐Occurrence Networks and Cluster Characteristics

We constructed microbial co‐occurrence networks for the rhizosphere and root compartment each using the SPIEC‐EASI method (Kurtz et al. 2015). Prokaryotes and fungi were incorporated together in each of the two networks (Tipton et al. 2018). We first excluded rare ASVs < 100 reads in total and ASVs that occurred in < 6 samples. Co‐occurrence networks were then calculated based on clr‐transformed read counts and the neighbourhood selection method with optimised stability parameters based on the StARS selection procedure (*n* = 94 *Festuca* plants; threshold 0.05, nlambda 45 with 300 replications) (Liu et al. 2010). Similarly responding ASVs in each network were clustered using the Spin‐glass algorithm of the igraph package (Newman and Girvan 2004; Reichardt and Bornholdt 2006; The igraph Core Team 2020; Traag and Bruggeman 2009). This method identifies clusters using both positive and negative edges between ASVs, without requiring a predefined number of clusters. Importantly, by clustering based on co‐occurrence network structure rather than, for example, assumed microbial function, this approach allowed us to detect ecological groupings that preserves the likely complex and divergent responses of microbial subgroups within communities.

For each cluster in each network, we summed the relative read counts of the ASVs per sample and tested for significant correlations between clusters using linear mixed effects models using lme of the nlme package (Pinheiro et al. 2019). Location was incorporated as a random effect in these models. Relative read counts of clusters were ln‐ or sqrt‐transformed in case model residuals did not follow a normal distribution. *p*‐Values were adjusted for multiple comparisons using the Bonferroni correction (Bonferroni 1936). Additionally, we tested temperature, precipitation, and their interaction (continuous variables) for each microbial network cluster using generalised linear mixed models with a beta distribution and location as a random effect using the glmmTMB package (Magnusson et al. 2017).

For each microbial cluster in the rhizosphere and root network we calculated a set of characteristics to infer its putative function. We calculated the average relative abundance of microbial phyla, classes, orders and families and, where possible, inferred putative characteristics of the cluster based on knowledge obtained from literature on the present microbial taxa (Tables S1 and S2). For each cluster, we calculated the average relative size and the average relative abundance of prokaryotes and fungi based on relative read counts (Tables S3 and S4).

We also calculated the number of unique prokaryote and fungal ASVs present in each cluster and defined whether the cluster held relative habitat generalists or specialists (Tables S3 and S4). For this, we calculated the community weighted mean of the SI of each sample in each cluster. Relative habitat generalist clusters were defined as clusters below the community‐wide mean SI, while relative habitat specialists were defined as clusters above the community‐wide mean SI (Chen et al. 2021). Clusters with an overlap in interquartile range of SIs with the community‐wide mean SI were defined as unspecified (Figure S2).

### 2.7 Soil Property Measurements

Soil collected from below each *F. rubra* was sieved on a 2 mm mesh and thoroughly mixed. Plant‐available nitrogen (N) (mg kg^−1^ dry soil) was measured by extracting N compounds using 50 mL of 0.5 M K_2_SO_4_ with 5 g of fresh soil. The mixture was shaken for 2 h after which the soil was filtered out. NO_3_^−^, NH_4_^+^ and NO_2_^−^ concentrations were determined in the filtrate by Flow Injection Analysis (QuickChem 8000 FIA; Lachat Instruments, Loveland, CO, USA). To determine plant‐available phosphorus (P), 5 g air dried soil was extracted with 50 mL of 1 M NaHCO_3_. The solution was adjusted to pH 8.5 by adding activated carbon to eliminate discoloration resulting from humic acid release. The solution was shaken for 30 min, soil was filtered out, and available P was determined in the filtrate by the Olsen photometric method (ATI Unicam UV 400/VIS Spectrophotometer at 630 nm) (Olsen et al. 1954). Soil pH was measured by shaking 5 g air dried soil with 25 mL deionised water for 30 min and measuring pH in the filtrate (WTW Multilab 540; Xylem Analytics, Weilheim, Germany). Soil total N and C content were determined using combustion analyses (FLASH 2000 CHNS/O Analyzer; Thermo Fisher Scientific, Waltham, MA, USA) on dried soil ground to < 0.1 mm particle size.

### 2.8 Start of the Growing Season, Temperature and Soil Moisture Calculations

At each location, the start of the growing season was calculated as the number of days between the first date in 2020 when soil temperatures rose above 2.5°C and our sampling date in summer. Mean winter temperature in the vegetation layer was determined as the 12‐year mean temperature at 30 cm above the soil during the winter period from January until March (soils began to thaw in April for the first time since winter). For each soil moisture sensor at each location, we calculated the 12‐year average daily mean soil moisture from 2009 until 2020. These values were averaged values across the two sensors per location. Circadian soil moisture fluctuation was determined by averaging soil moisture levels for each hour across all years and calculating the coefficient of variation over this 24 h period (Figure S3). In cases where soil moisture or temperature sensors failed for more than 5 months in a given year, the affected year was excluded from the calculation.

We tested whether the 12‐year mean values for average winter temperature in the vegetation, average soil moisture, and circadian soil moisture fluctuation showed significantly different patterns compared to the values calculated for the year 2020 alone. Since no significant differences were found, we chose to use the 12‐year mean values, as these provided averages with lower error rates (data not shown).

### 2.9 Plant Community Composition

Plant species cover data was obtained from control plots of a climate change transplant experiment by Vandvik et al. (2020) and Lynn et al. (2021). At each location, five 25 × 25 cm plots provided plant community cover data on a plant species level, of which we used data from 2019 only. For each plant species occurring in the plot, the cover was visually estimated and plant community diversity was calculated for each location using the Shannon diversity index and vegan package (Oksanen et al. 2018). Cover estimates were summed per functional plant group (forbs, grasses excluding *F. rubra* , legumes, shrubs, pteridophytes, hemiparasites, and sedges and rushes together) and *F/rubra* . Total bryophyte cover and soil litter cover were obtained from measurements in the same plots obtained in 2013, when a detailed study of bryophytes and litter cover in the plots was conducted (Pascal et al. unpublished) (Klanderud et al. 2015; Vandvik et al. 2020). We consider the combination of data from different years to be justified, because of the generally slow growth of bryophytes in cold climates and the minimal shifts in plant community composition that were previously observed over time (Lynn et al. 2021; Vandvik et al. 2020).

### 2.10 Structural Equation Models

We hypothesised that temperature and precipitation affected plant–soil microbiota interactions directly, were mediated by changes in the start of the growing season, via plant community composition and/or through soil abiotic properties. We used structural equation models (SEM) to separate these different pathways that may play a role in mediating temperature and precipitation effects. All SEMs were fit using piecewiseSEM (Lefcheck 2016) and lme of the nlme package (Pinheiro et al. 2019) with location as random effect.

To minimise model complexity upfront, we adopted a two‐step approach following in 't Zandt et al. (2023). First, we calculated a base model (the upstream part of the model) defining the significant pathways via which temperature and precipitation affect the start of the growing season, winter temperature in the vegetation, soil moisture variables, plant community composition, and soil abiotic properties (i.e., microbial data were excluded in this first step). This approach is valid because effects in the model are one‐directional, meaning that additional variables can be appended downstream without affecting the upstream parts of the model. Additionally, we focused only on pathways directly related to temperature and/or precipitation, and excluded climate‐unrelated pathways. Furthermore, we simplified our models using a backward stepwise elimination procedure for which we consecutively removed pathways with the highest *p*‐value and tested whether pathway removal improved model fit (in 't Zandt et al. 2020). The model with the lowest Akaike information criterion (corrected for small sample sizes; AICc) was selected as the best fit base model. Endogenous variables were allowed to drop from the model in case effects were not significant (*p* > 0.05). Additionally, overall model fit was assessed using directional separation tests (d‐sep) based on Fisher's C statistics with models being accepted if *p* > 0.05.

Next, each microbial network cluster (summed relative reads per rhizosphere and root microbial network cluster from the calculated co‐occurrence networks; 16S and ITS ASVs separately), as well as alpha‐diversity indices and average SI of the microbial communities were ran through the SEM model as the final parameter to be estimated. Per run, one microbial parameter was considered, which could be affected either directly by temperature and precipitation and/or indirectly via the start of the growing season, plant community composition and/or soil abiotic properties. Each run, a backward stepwise elimination procedure to consecutively remove non‐significant pathways was followed in the same way as performed for the base part of the SEM. All microbial variables not following a normal distribution were ln‐ or sqrt‐transformed and location was taken into account as a random factor.

### 2.11 Calculation of Explained Variation Microbial Co‐Occurrence Networks

In total, we created 34 unique SEM models based on the microbial co‐occurrence networks: 7 for 16S rhizosphere network clusters, 7 for ITS rhizosphere network clusters, 11 for 16S root network clusters, and 9 for ITS root network clusters. From each mode, we extracted the effect sizes of each significant pathway (*p* < 0.05). For each significant pathway, we calculated composite effect sizes by multiplying the effect sizes between intermediate variables, where applicable. We summed the effect sizes of all significant pathways affecting a microbial network cluster for each climate, plant, and soil variable (in 't Zandt et al. 2023). These were then scaled to the relative size of the involved network clusters and the percentage of explained variation of the SEM for the involved network cluster.

## 3 Results

### 3.1 Temperature and Precipitation Shape the Rhizosphere and Root Microbiome

**FIGURE 1:**
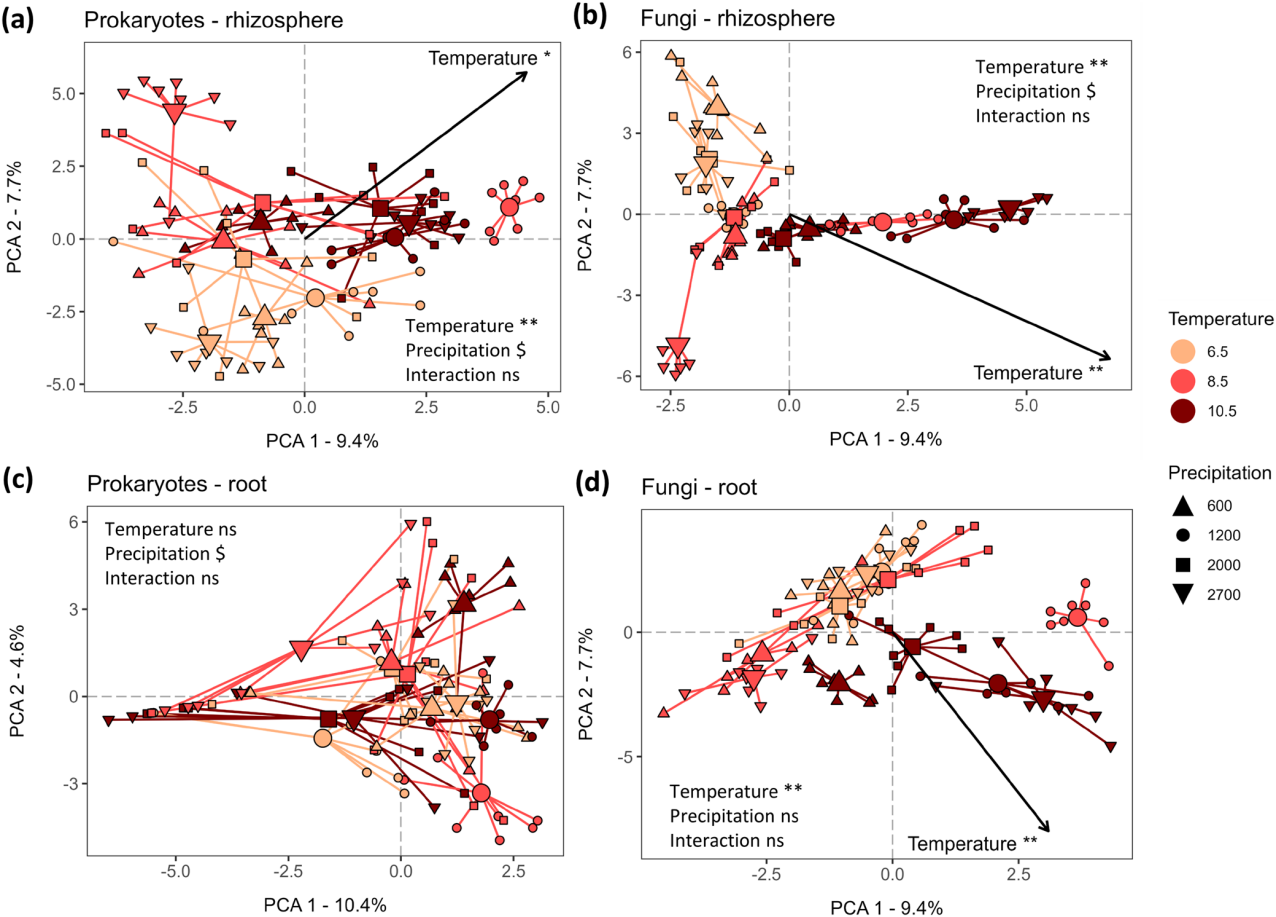
Principal Component Analysis (PCA) illustrating temperature and precipitation effects on plant–soil microbiota associations. (a) Rhizosphere prokaryotes, (b) rhizosphere fungi, (c) root‐associated prokaryotes, and (d) root‐associated fungi of *Festuca rubra* . Colours indicate the temperature gradient (average summer temperature in degrees Celsius) and shapes the precipitation gradient (average annual precipitation in mm). Small shapes indicate the position of each *F. rubra* individual and large shapes indicate the centroids of each location (*n* = 8 rhizosphere and root samples per location with 12 locations in total). Results of PERMANOVA tests on significant separation of temperature, precipitation, and their interaction are presented (based on tests run on sample centroids). Arrows represent the passive projection of the temperature gradient, showing its significant relationship with the first two PCA axes (based on tests using sample centroids; precipitation was not significantly related to these first two axes). Significance codes: **0.01 < *p* < 0.01; *0.01 < *p* < 0.05; ^$^0.05 < *p* < 0.1.

Rhizosphere and root‐associated prokaryote and fungal communities of *Festuca rubra* showed significant separation between the 12 locations (Figure 1a–d). Separation of rhizosphere communities was significantly related to temperature and marginally significantly related to precipitation. Separation of root‐associated prokaryote communities was only marginally related to precipitation, and root‐associated fungal communities were only significantly related to temperature (Figure 1a–d).

Prokaryote communities were highly unique between the rhizosphere and root compartments. Only 17% of prokaryote taxa were observed in both the rhizosphere and root compartments (Figure S4a,b). Within each compartment, however, a large core community of prokaryotes was present. 84% and 79% of prokaryote taxa were present at all three temperature levels, and 77% and 63% at all four precipitation levels in the rhizosphere and root compartments, respectively (Figures S5a,b and S6a,b).

Fungal communities, on the other hand, showed an overlap of 85% in taxa occurrence between the rhizosphere and root compartments (Figure S4a,b). Fungal taxa occurrence was more variable than for the prokaryote community, with only 33% and 49% present at all three temperature levels, and 26% and 38% at all four precipitation levels for the rhizosphere and root compartments, respectively (Figures S5c,d and S6c,d).

### 3.2 Microbial Co‐Occurrence Networks Were Affected by Temperature, Precipitation, and Their Interaction

**FIGURE 2:**
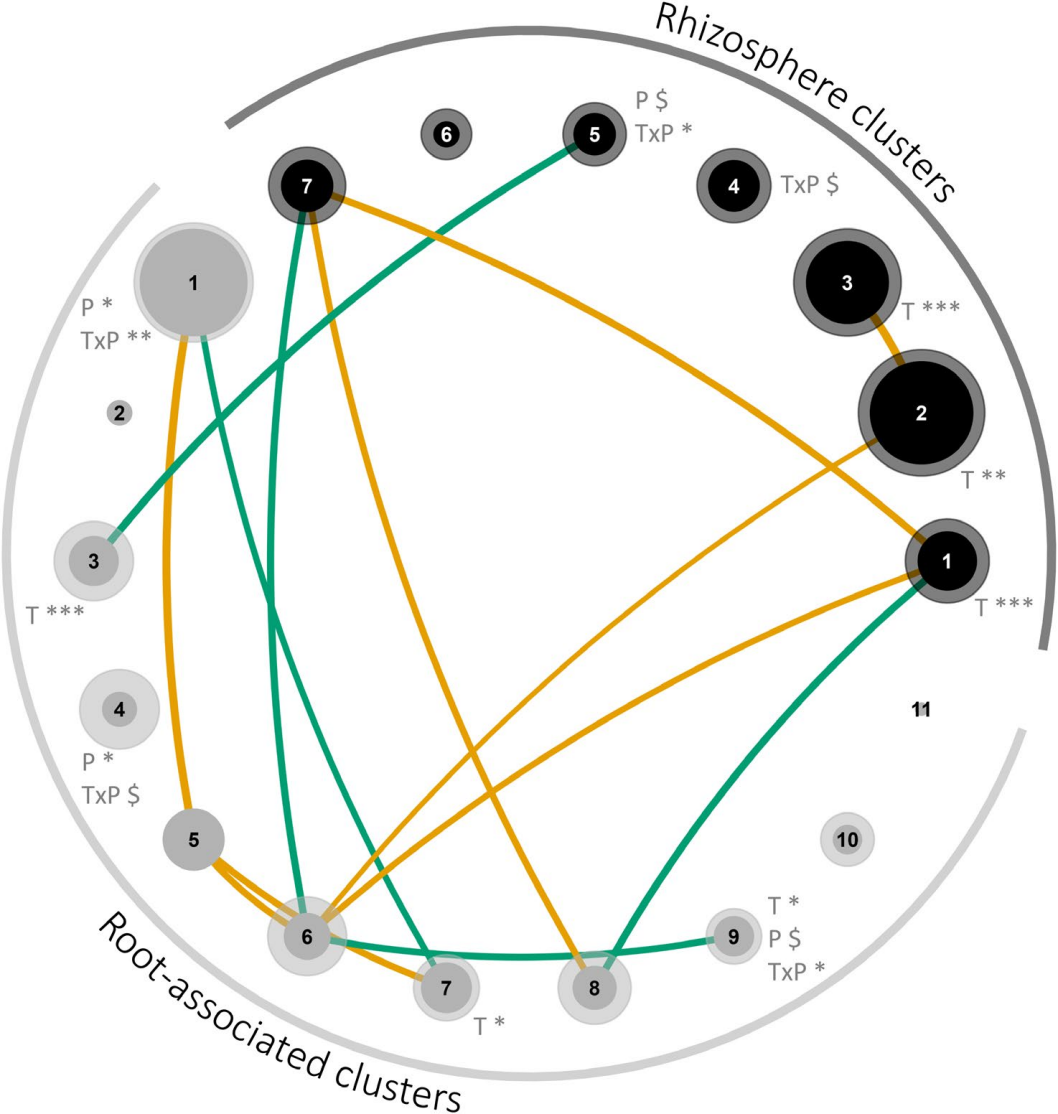
Co‐occurrence network clusters of rhizosphere (dark grey) and root (light grey) microbial communities. Solid, central zones of each circle indicate the average relative abundance of prokaryote taxa in the cluster. Transparent, outer zones of each circle indicate the average relative abundance of fungal taxa in the cluster. Root‐associated clusters 2, 5 and 11 contain prokaryote taxa only. Significant correlations between clusters are indicated by lines between clusters. Negative correlations are indicated in yellow, positive correlations in green. Width of lines indicate the strength of the correlation. Significant and marginally significant effects of temperature (T), precipitation (P) and their interaction (TxP) are indicated next to each cluster. In all statistical models, location was taken into account as a random effect. Significance codes: ***p < 0.001; **0.001 < *p* < 0.01; *0.01 < *p* < 0.05; ^$^0.05 < *p* < 0.07. For full networks, see Figure S7, for taxonomic and putative functions of each microbial network cluster see Figure S8 and Tables S1, S2, and for full statistical results, see Tables S5 and S6.

We created microbial co‐occurrence networks for the rhizosphere and root compartments separately. In each network, we identified network clusters, which grouped similarly responding prokaryote and fungal taxa (Figures 2, S7 and S8). Importantly, both the rhizosphere and root co‐occurrence networks were significantly more densely clustered than randomised networks, showing that a distinct organisational structure occurred in both rhizosphere and root‐associated microbial networks (Figure S9).

Microbial co‐occurrence networks in the rhizosphere were grouped into seven clusters, while in the roots 11 clusters occurred (Figures 2, S7a and S8a,b). Temperature, precipitation, and their interaction significantly affected microbial clusters in both the rhizosphere and root (Figure 2; Tables S5 and S6). All clusters in the rhizosphere network contained both prokaryote and fungal taxa, showing that prokaryotes and fungi responded relatively similarly within the rhizosphere compartment (Figures 2, S7a and S8a,b). In the root network, various microbial clusters were dominated by either prokaryotes or fungi, indicating that the responses of prokaryotes and fungi were in part decoupled within the root compartment (clusters 2, 5, 11; Figures 2, S7b and S8c,d). Between the rhizosphere and root network clusters, we observed only three positive correlations, showing that the microbial networks in these two compartments largely showed different patterns and were thus decoupled (Figure 2).

Where possible, we characterized the putative functions that could be performed by each network cluster (Tables S1 and S2). Most of the rhizosphere network clusters harbored taxa potentially involved in organic matter degradation and N‐cycling (Table S1). In contrast, the majority of root clusters harbored taxa potentially involved in plant pathogen attack, the suppression of plant diseases, and the degradation of organic matter (Table S2).

### 3.3 Temperature and Precipitation Separately as Well as Jointly Shape Plant Community Composition and Soil Properties

**FIGURE 3:**
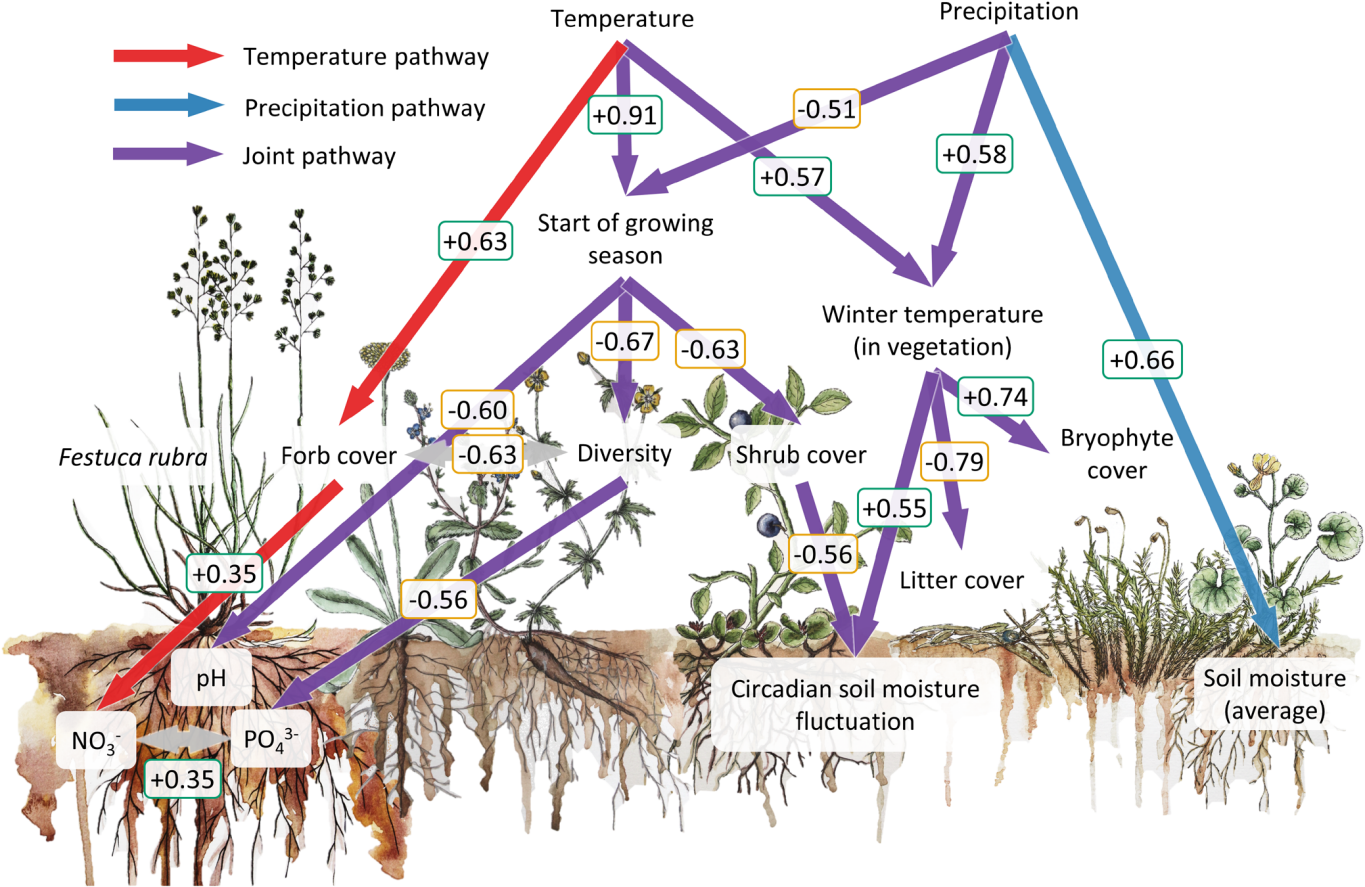
Structural equation model (SEM) showing effects of temperature, precipitation and their joint pathways on plant community composition, bulk soil properties (circadian soil moisture fluctuation and average soil moisture) and local *Festuca rubra* soil properties (NO_3_^−^, PO_4_^3−^, and pH). Red arrows indicate significant pathways regulated by temperature only, blue arrows indicate pathways regulated by precipitation only, and purple arrows indicate pathways regulated by both temperature and precipitation (joint pathways) (*p* < 0.05). Grey arrows indicate significant correlations (*p* < 0.05). Numbers indicate pathway effect sizes ranging from −1 (negative effects; yellow) to +1 (positive effects; green) (*n* = 12–94 over 12 locations). Absence of arrows indicates that pathways were not significant (*p* > 0.05).

To understand via which pathways temperature and precipitation shaped the rhizosphere and root microbial communities, we traced these climate effects through the plant and soil ecosystem using structural equation modelling (SEM). We found that both temperature and precipitation, separately as well as jointly, had a profound impact on plant community composition, which cascaded into affecting local *F. rubra* soil properties (Figure 3).

For both temperature and precipitation, a unique pathway occurred where the two factors operated independently. Precipitation increased average soil moisture (Figure 3), while temperature increased forb cover and thereby mediated a high availability of NO_3_^−^ in soil below *F. rubra* plants at warm locations (Figure 3).

In contrast, the start of the growing season and average winter temperature in the vegetation canopy were mediated by additive effects of temperature and precipitation. Specifically, high temperature advanced the start of the growing season, while high precipitation delayed the start of the growing season (Figure 3). The temperature in the vegetation canopy during the winter months was increased by both high temperature and high precipitation (Figure 3).

An early start of the growing season decreased soil pH below *F. rubra* plants, community shrub cover, and plant community diversity. The latter subsequently decreased PO_4_^3−^ availability below *F. rubra* plants, while decreased shrub cover increased the circadian soil moisture fluctuation at the location (Figure 3). This circadian fluctuation indicates that at low shrub cover, soil moisture dropped during the daytime, while at high shrub cover, soil moisture was constant during the day‐night cycle (Figure S3). Circadian soil moisture fluctuation was further increased by a high temperature during the winter months. A high winter temperature in the vegetation canopy furthermore increased bryophyte cover, while decreasing litter cover (Figure 3). *F. rubra* cover was not significantly affected by temperature or precipitation (data not shown).

### 3.4 Joint Pathways of Temperature and Precipitation Were Key in Shaping Microbial Co‐Occurrence Networks

**FIGURE 4:**
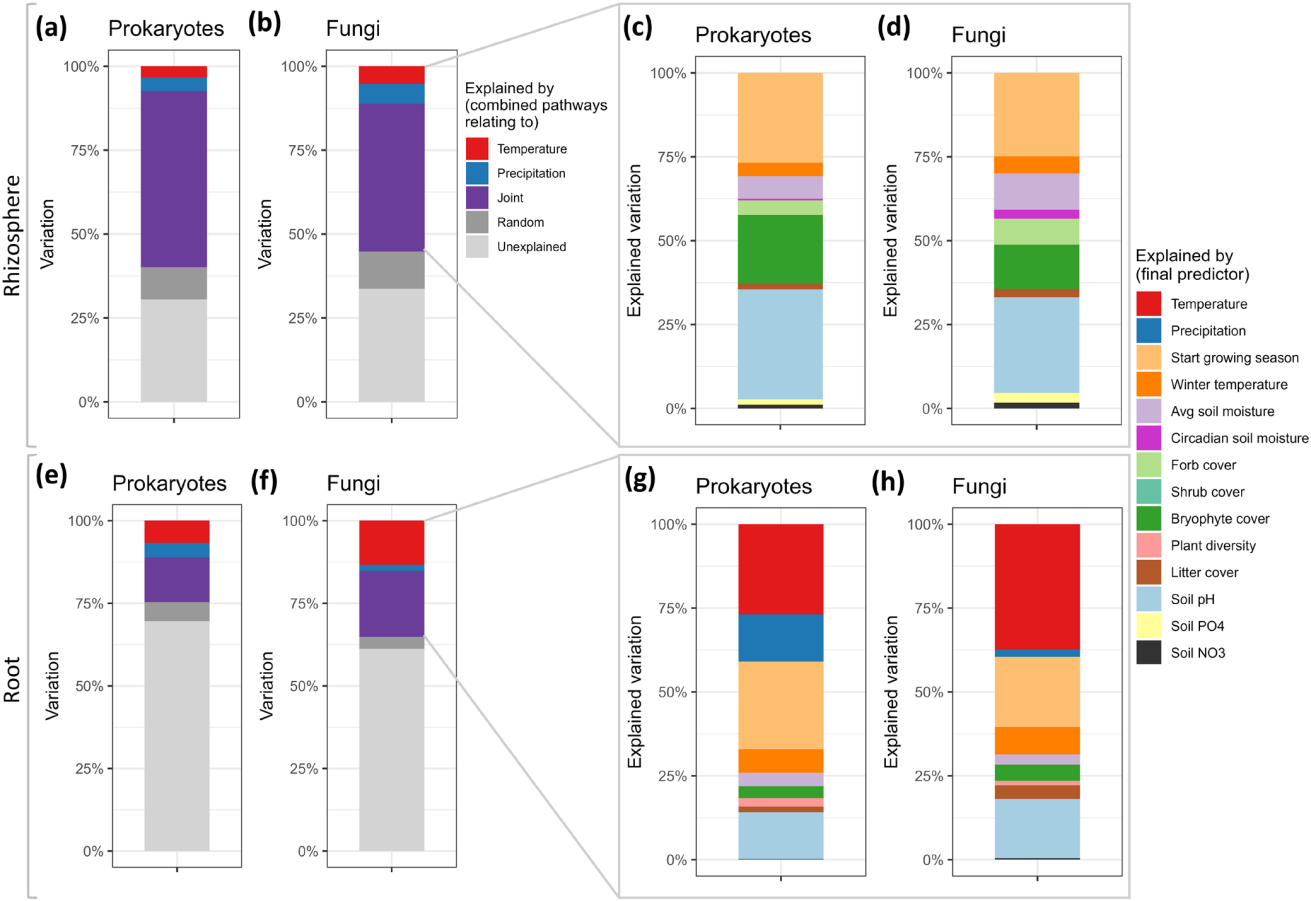
Relative contribution of temperature, precipitation and their joint pathways on (a–d) rhizosphere and (e–h) root‐associated microbial communities of *Festuca rubra* . In a, b, e, and f the combined relative contribution of all temperature, precipitation, and joint pathways in explaining variation in the rhizosphere and root‐associated microbial communities are shown. Panels c, d, g, and h zoom in on the explained variation from the respective full overview from a, b, e, or f, separated into the various underlying pathways (see Figure 3 for pathways). Relative contribution of pathways is based on the effect sizes of the structural equation model pathways, which were weighted by the size of the involved microbial rhizosphere or root cluster. Random refers to the relative contribution of within‐location variation that was taken into account as a random effect in each pathway. *N* = 94 *F. rubra* individuals distributed over 12 locations.

We determined via which pathways temperature and precipitation shaped the microbial rhizosphere and root network clusters by combining our SEM model with the microbial network clusters. In the rhizosphere, temperature, precipitation, and their joint pathways explained between 50% and 60% of the variation in prokaryote and fungal network clusters (Figure 4a,b). Most of this variation was explained by pathways jointly affected by temperature and precipitation (Figure 4a,b). The start of the growing season, bryophyte cover, and soil pH contributed most strongly to shaping rhizosphere prokaryote and fungal network clusters (Figure 4c,d). Especially for fungal network clusters, average soil moisture and forb cover contributed to a lesser, yet significant amount of variation (Figure 4c,d).

In contrast to the rhizosphere, only 25%–35% of variation in root prokaryote and fungal network clusters was explained by temperature, precipitation, and their joint effects (Figure 4e,f). Especially for fungal root‐associated network clusters, pathways shaped by temperature alone played a significant role (Figure 4e,f), while for root‐associated prokaryote network clusters, direct effects of precipitation explained more variation than in the rhizosphere (Figure 4g,h). Yet, similar to the rhizosphere, a large fraction of the explained variation was linked to pathways jointly affected by temperature and precipitation (Figure 4e,f). In line with the rhizosphere networks, the start of the growing season and soil pH were important pathways shaping microbial network clusters in the root (Figure 4g,h).

### 3.5 Temperature and Precipitation Affected Putative Organic Matter Degraders, Plant Pathotroph‐Saprotrophs, and Plant Beneficial Microbiota

We identified the microbial clusters affected by the most prominent temperature and precipitation pathways (Tables S1 and S2). In the rhizosphere, an advancement of the start of the growing season and subsequent drop in soil pH was associated with a decrease in relative habitat specialist prokaryotes and fungi. These prokaryotes and fungi were potentially involved in organic matter degradation, including the degradation of recalcitrant C sources (cluster 2 in Table S1). Simultaneously, distinct clusters of microbiota also potentially involved in degrading recalcitrant C sources were decreased (clusters 3 and 7 in Table S1). The latter group involved microbiota suggested to be facultatively anaerobic (cluster 3 in Table S1). In the root, an early start of the growing season increased mostly putative plant pathotroph‐saprotrophs and bacteria potentially promoting plant growth (clusters 2 and 3 in Table S2). Additionally, via the drop in soil pH with an early start of the growing season, mainly the putative fungal organic matter degrading community shifted in composition (clusters 6, 8, and 10 in Table S2).

In the rhizosphere, bryophyte cover decreased mainly relative habitat generalist microbiota potentially degrading recalcitrant C sources (clusters 2 and 3 in Table S1). In contrast, forb cover, soil moisture, and litter cover increased fungi and relative habitat specialists prokaryotes potentially involved in organic matter degradation (clusters 4, 5, and 7 in Table S1). In the root, direct temperature effects decreased putative organic matter degrading microbiota (cluster 8 in Table S2), while precipitation decreased relative habitat generalist microbiota characterized mostly as putative plant pathogens and beneficial microbiota (cluster 1 in Table S2).

### 3.6 Contrasting Effects of Temperature and Precipitation on Habitat Specialisation of Microbiota in the Rhizosphere and Root

**FIGURE 5:**
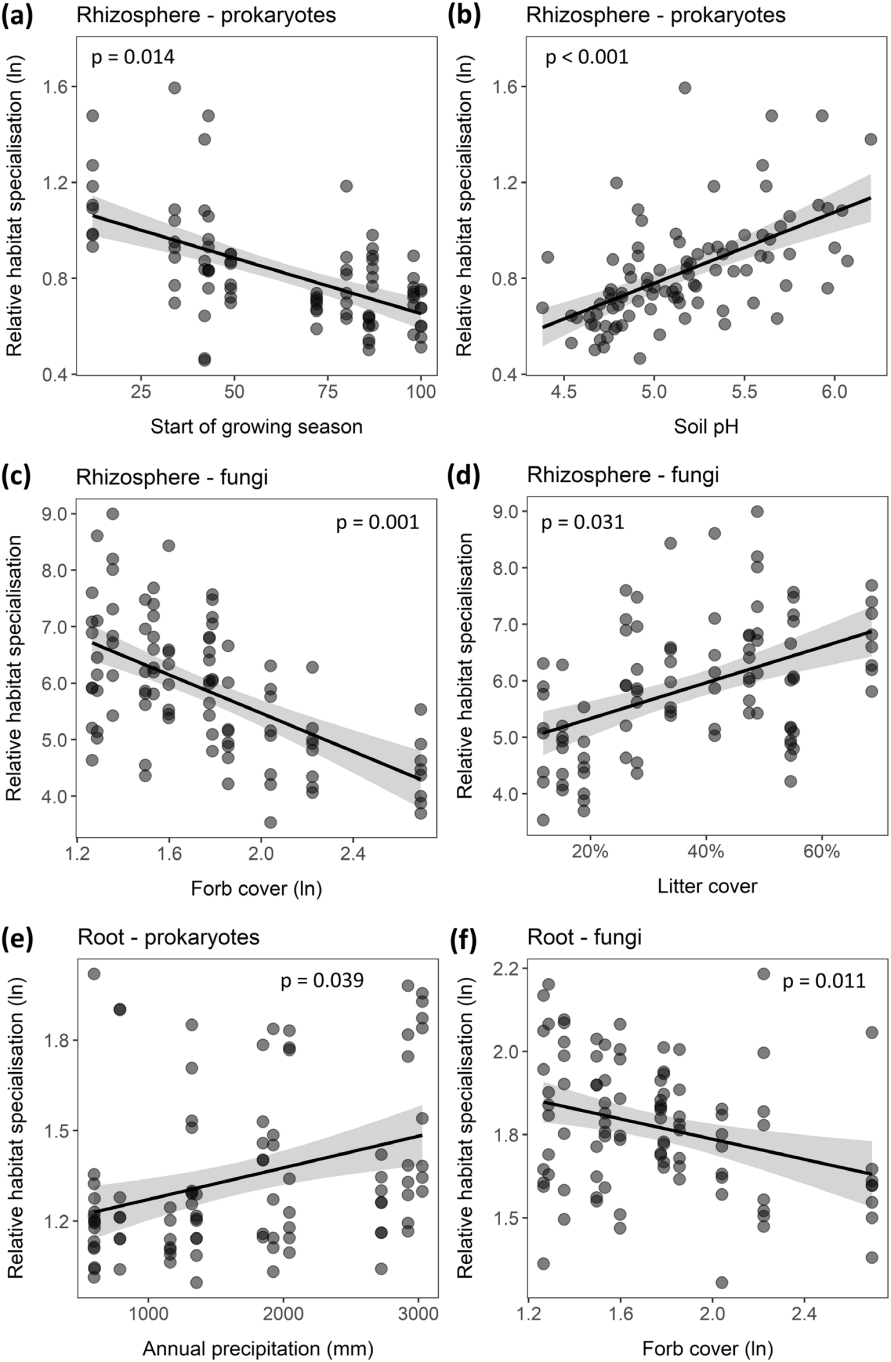
Significant relations between relative habitat specialisation of (a–c) rhizosphere microbiota and (d–f) root‐associated microbiota to climate, community vegetation cover and soil properties. The start of the growing season is indicated as the number of days after soil defrosting. Solid lines indicate the mean relation with in grey the 95% confidence interval. Relative habitat specialisation is the community weighted mean of the specialisation index of all ASVs present in a sample. Higher values indicate a greater relative habitat specialisation of the microbial community. Statistical results of the pathways from structural equation models are shown (*n* = 94 *F. rubra* individuals distributed over 12 locations with location taken into account as a random effect).

Finally, on an overall microbial community scale, we found that increasing temperature decreased and increasing precipitation increased relative habitat specialisation of the prokaryote community. These effects occurred via advances and delays in the start of the growing season and subsequent effects on soil pH (Figures 3 and 5a,b). Relative habitat specialisation of the rhizosphere fungal community, on the other hand, was decreased with temperature via an increase in forb cover (Figures 3 and 5c), and further decreased by both temperature and precipitation via a reduction in litter cover with higher winter temperatures in the vegetation (Figures 3 and 5d). Only the relation between prokaryotes and soil pH was, in part, related to changes in prokaryote Shannon diversity (Figure S10).

In the root‐associated compartment, precipitation directly increased relative habitat specialization of the prokaryote community (Figure 5e). Relative habitat specialization of the root‐associated fungal community was, similar to the rhizosphere fungal community, decreased by increasing temperature via an increase in forb cover (Figures 3 and 5e). None of these patterns were driven by differences in Shannon diversity (data not shown).

## 4 Discussion

### 4.1 The Start of the Growing Season Mediates Joint Effects of Temperature and Precipitation on Plant–Soil Microbiota Interactions

In alpine grassland communities, we tested via which pathways temperature and precipitation shape plant–soil microbiota interactions. Our findings show that joint pathways of temperature and precipitation are most critical in shaping the rhizosphere and, to a lesser extent, the root‐associated microbiome of *F. rubra* than temperature and precipitation pathways alone. This aligns with Münzbergová et al. (2017), who demonstrated that both temperature and precipitation are important in determining the growth strategy of *F. rubra* , such as determining its investment in rhizome and root biomass. Similarly, combined effects of temperature and precipitation were found to drive plant community productivity as well as plant species colonization and extinction rates within the same climate grid (Klanderud et al. 2015; Meineri et al. 2013; Vandvik et al. 2020).

We further found that the joint effects of temperature and precipitation on plant‐microbiota interactions were primarily mediated by changes in the start of the growing season and subsequent effects on soil pH. Specifically, higher temperatures advanced the start of the growing season, leading to a decrease in soil pH. In contrast, higher precipitation delayed the start of the growing season, resulting in an increase in soil pH. These opposing effects likely result from the contrasting effects of temperature and precipitation on snow conditions during winter (Yu et al. 2013). Higher temperatures lead to quicker snowmelt, advancing the start of the growing season, while increased precipitation leads to greater snow cover, extending snow cover duration and delaying the start of the growing season (Kumar et al. 2012; Rebetez 1996; Yu et al. 2013).

Indeed, soil microbial communities undergo marked shifts between winter and summer (Bardgett et al. 2005; Broadbent et al. 2021), and snow removal experiments show that an advancement of the start of the growing season significantly reshapes soil microbial communities and reduces soil pH (Broadbent et al. 2021, 2024; Gavazov et al. 2017). This reduction in soil pH is likely related to an earlier onset of biological activity triggered by an earlier start of the growing season. This would lead to increased plant root exudation, microbial nitrification, and organic matter turnover, which are all processes that generate acidity and thus reduce soil pH (De Boer and Kowalchuk 2001; Jones et al. 2004; Wang and Kuzyakov 2024b). We conclude that in cold environments, the start of the growing season is a critical factor mediating joint effects of temperature and precipitation on plant–soil microbiota associations.

Our findings suggest that in locations where both temperature and precipitation are increasing due to climate change, their opposing effects on the start of the growing season may moderate changes in plant–soil microbiota associations. In contrast, in locations where temperature is rising and precipitation is decreasing, both factors advance the start of the growing season, likely amplifying each other's effect on plant–soil microbiota associations. We therefore suggest that in overall cold and wet environments, shifts in plant–soil microbiota associations are particularly accelerated in areas experiencing both warming and drying. At the same time, given the critical role of winter snow cover in these interactions, it is important to note that climate change is causing more winter precipitation to fall as rain (Mankin and Diffenbaugh 2015). The transition from snow to rain during winter therefore represents a critical tipping point, likely to further accelerate changes in plant–microbiota interactions.

### 4.2 An Early Start of the Growing Season Increased Habitat Generalist Rhizosphere Prokaryotes and Putative Pathotroph‐Saprotrophs in the Root

Changes in the start of the growing season affected rhizosphere and root‐associated prokaryotes and fungal communities. An early start of the growing season and subsequent drop in soil pH increased the abundance of habitat generalist prokaryotes in the rhizosphere. Prokaryotes become active after snowmelt when a surge in available N in the soil occurs, and exhibit a fast turnover throughout the season (de Vries et al. 2018; in 't Zandt et al. 2023; Oram et al. 2023). Hence, rhizosphere prokaryotes specialized to winter conditions were likely outcompeted as time passed since the start of the growing season, explaining the observed increase in habitat generalists in the rhizosphere.

Many rhizosphere microbiota and root‐associated fungi affected by the start of the growing season were likely involved in degrading recalcitrant C sources. From a seasonal perspective, this involvement makes sense, as labile C compounds that accumulate in the soil during autumn and winter are rapidly degraded early in the season following snowmelt. This process leaves primarily recalcitrant compounds available in summer when we sampled (Bardgett et al. 2005; Broadbent et al. 2021). These patterns also suggest high redundancy within the microbial community affected by the start of the growing season, because microbiota performing similar functions appeared to replace one another in response to shifts in the start of the growing season. Consequentially, the functional impact of climate change through shifts in the start of the growing season on rhizosphere microbiota and root‐associated fungi may be relatively limited.

In contrast to decomposer taxa, functional redundancy was not evident among the putative fungal pathotroph‐saprotrophs and beneficial bacteria in the root that were affected by the start of the growing season. Both groups increased in relative abundance with an early start of the growing season, possibly resulting from a longer period for the accumulation of specific plant‐microbiota interactions to occur. Given that the most commonly observed effect of rising temperatures is an increase in plant disease (Singh et al. 2023) and various of the involved fungi are best known for their plant pathogenic behavior (Chen et al. 2016; Crous et al. 2014), these findings suggest that plant pathogen pressure, along with a compensatory increase in beneficial bacteria, may have occurred with an earlier onset of the growing season due to warming. At the same time, root‐associated fungi are well known to switch between pathotrophic and saprotrophic lifestyles in relation to plant growth stages and environmental changes (Liao et al. 2025), and fungal saprotrophs often respond to similar environmental and host plant cues as plant pathogens (Li et al. 2024; S. Liu et al. 2023), explaining why we observed concomitant shifts within these two groups. We suggest that shifts in the growing season start play a critical role in reshaping the functioning of plant root microbiomes, potentially increasing plant disease pressure with the ongoing change in climate, alongside concurrent shifts in microbiota involved in decomposition. These findings demonstrate both the broad and complex ecological consequences of altered growing season dynamics with the ongoing change in climate in cold environments.

### 4.3 Temperature and Precipitation Increased Habitat Generalist Plant‐Fungal Interactions

Where habitat specialization for the rhizosphere prokaryote community was related to changes in the start of the growing season, different pathways were at play for the rhizosphere and root‐associated fungal communities. In both the rhizosphere and root compartments, higher temperatures and increased precipitation led *F. rubra* to associate with more fungal habitat generalists. In both compartments, these patterns resulted from an increase in forb cover with temperature. Additionally, in the rhizosphere, temperature and precipitation increased winter temperatures in the vegetation and subsequently reduced litter cover, which also contributed to an increase in fungal habitat generalists. This increase in generalists suggests a loss of heterogeneity in plant‐associated fungal communities as well as a shift towards communities that may be more resistant to environmental change at the individual taxon level. This is because habitat generalists, with their broad environmental tolerance, are more resilient to environmental change, while habitat specialists, with narrower environmental ranges, are more vulnerable to these changes (Arraiano‐Castilho et al. 2021; Chen et al. 2021). However, from a whole community perspective, such shifts are likely destabilizing. Specialized interactions between plants and soil microbiota, as well as spatial heterogeneity in the soil, are key drivers of plant species coexistence, community diversity, and long‐term stability (in 't Zandt et al. 2021, 2022, 2023; van der Putten et al. 2016). Combined with the high resistance of habitat generalists to change, these observed shifts are likely to be difficult to reverse, if not irreversible. We conclude that in cold regions where both temperature and precipitation are increasing, specialist interactions between plants and soil fungi are being lost, which is likely to significantly alter the mechanisms structuring ecosystem dynamics.

Interestingly, the percentage cover of *Festuca rubra* was not affected by temperature or precipitation, showing the true generalist nature of our host plant species. As a consequence, loss of specialist plant‐microbiota interactions may be less problematic for such a generalist species, compared to specialist host plants that often rely on specific microbial partners for their growth and survival. For such plant species, changes in climate and loss of specialist microbiota may thus be more adverse, ultimately leading to reduced fitness or shifts in plant species competitive dynamics (Hawkes et al. 2020).

Many fungal rhizosphere and root taxa were potentially involved in organic matter degradation, including those related to changes in litter and forb cover. Indeed, many fungi specialize in degrading recalcitrant plant litter (Bardgett et al. 2005), explaining why fungal habitat specialists decreased with a reduction in litter cover. Additionally, the decline of specialist fungi with increased plant community forb cover due to rising temperatures is likely related to the capacity of acquisitive forb species to promote the turnover of soil organic matter by exuding labile C compounds in their rhizosphere (Henneron et al. 2020). This exudation of labile compounds likely favored fast‐growing, habitat generalist microbiota over fungi specialized in degrading recalcitrant organic matter. We conclude that in cold areas experiencing increases in both temperature and precipitation, the degradation of recalcitrant organic matter by plant‐associated fungi will increasingly be performed by habitat generalists.

In contrast to the fungal community, the root‐associated prokaryote community was increased in habitat specialists with high precipitation. This effect likely resulted from more frequent soil anoxic conditions due to high precipitation, along with associated changes in plant physiology and root traits (Hartman et al. 2019; Oram et al. 2020, 2021; Parent et al. 2008). These conditions namely favor specialist prokaryotes that can tolerate or thrive in this specific environment (Chen et al. 2021). This selective pressure was likely experienced to a lesser extent by the fungal community, as fungi are generally more resistant to environmental disturbances than prokaryotes (de Vries et al. 2018; in 't Zandt et al. 2023; Oram et al. 2023). The prokaryotes affected by precipitation included putative plant pathogens and beneficial bacteria. Our findings therefore suggest that an increase in precipitation in cold environments will result in plant disease dynamics being increasingly regulated by habitat specialist prokaryotes.

### 4.4 Bryophytes Are a Key Mediator of Changes in Temperature and Precipitation

Bryophytes have been shown to be key in regulating soil microclimates and plant species invasion success (Jaroszynska et al. 2023; Vandvik et al. 2020). In line with the current study, we found that bryophyte cover was a critical factor in decreasing the abundance of microbiota involved in organic matter degradation in the rhizosphere of *F. rubra* . This effect was enhanced by both temperature and precipitation and was mediated by an increase in winter temperature in the vegetation layer. Climate change increases both summer and winter temperature, while precipitation increases winter temperature in the vegetation by enhancing snow cover, which insulates the vegetation layer (Y. Liu et al. 2023). Higher winter temperatures and sustained soil moisture due to snow cover likely increased bryophyte cover (Cooper et al. 2019; Górski et al. 2020). Bryophytes then acted as thermal insulators for the soil, reducing diurnal soil temperature fluctuations (Jaroszynska et al. 2023; Lindo et al. 2013). This higher and more constant soil temperature may have accelerated soil microbial processes, thus decreasing slow‐growing rhizosphere microbiota degrading recalcitrant C sources. We conclude that bryophytes play a crucial role in mediating the effects of temperature and precipitation change on plant–soil microbiota interactions.

## 5 Conclusion

We demonstrate that in cold environments, the onset of the growing season is mediated by joint effects of temperature and precipitation, and therewith key in shaping interactions between plants and soil microbiota. Temperature and precipitation had opposing effects on the onset of the growing season, likely due to their opposing effects on snow cover duration. These findings highlight the complexity of plant–soil microbial interactions in response to climate and underscore the necessity for local and seasonal monitoring of climatic variables including snow cover to accurately predict ecosystem responses to climate change.

We found that soil pH and bryophyte cover explained a high proportion of the variation in plant rhizosphere and root microbiomes related to temperature and precipitation gradients. An early start of the growing season reduced soil pH, while increasing temperature and precipitation both enhanced bryophyte cover. These two variables thus indicate shifts in plant–soil microbiota interactions and may serve as easily measurable indicators for monitoring ecosystem responses to the changing climate.

Our findings further suggest that with rising temperature and precipitation in cold grassland regions, plant‐fungal interactions become more tolerant to a wider range of environmental conditions and thus lose specificity, while root prokaryotes gain more specialists. Changes in temperature and precipitation mainly shifted microbial communities performing soil organic matter turnover and putative plant pathogens. These findings indicate that the plant–soil microbiota interactions that structure plant community dynamics are significantly changing with the ongoing climate change (Aldorfová et al. 2020; Bever et al. 2012; Florianová and Münzbergová 2025; in 't Zandt et al. 2021, 2022; Kardol et al. 2006; Semchenko et al. 2019; van der Putten et al. 2016). We conclude that limiting temperature increase and precipitation change is critical to safeguarding the unique plant‐microbiota interactions that shape the functioning of cold climate systems.

## Author Contributions

**Dina in 't Zandt:** conceptualization, data curation, formal analysis, methodology, software, visualization, writing – original draft, writing – review and editing. **Anna Florianová:** investigation, writing – review and editing. **Mária Šurinová:** investigation, methodology. **Michiel H. in 't Zandt:** formal analysis, writing – review and editing. **Kari Klanderud:** conceptualization, investigation, writing – review and editing. **Vigdis Vandvik:** conceptualization, funding acquisition, investigation, project administration, writing – review and editing. **Zuzana Münzbergová:** conceptualization, funding acquisition, project administration, supervision, writing – review and editing.

## Conflicts of Interest

The authors declare no conflicts of interest.

## Supporting information


**Data S1:** gcb70431‐sup‐0001‐Supinfo.docx.

## Data Availability

All data generated in this study are publicly available via the Zenodo digital data repository at https://zenodo.org/records/16409277. Microbial sequencing data generated in this study are deposited in the NCBI SRA database in BioProject PRJNA1295349 at https://www.ncbi.nlm.nih.gov/sra/PRJNA1295349. Vegetation, litter, soil moisture, soil temperature, and air temperature data are publicly available from the Vestland Climate Grid OSF data repository at https://doi.org/10.17605/OSF.IO/NPFA9. Fungal trait data is publicly available via the FungalTrait database at https://doi.org/10.1007/s13225‐020‐00466‐2. All R scripts are publicly available via Github at https://github.com/dintzandt/Norway_temperature_precipitation_rhizosphere_root_microbiome and the Zenodo digital data repository at https://zenodo.org/records/16737047.
